# Additive effect of polyethylene-polypropylene fibers in heat-cure acrylic resin flexural strength

**DOI:** 10.6026/973206300214767

**Published:** 2025-12-15

**Authors:** Bodduluri Manisha, Ravi Kumar C, Sujesh M, Rajanikanth A.V, Harilal G, Kavitha Ch

**Affiliations:** 1 Department of Prosthodontics and crown and bridge, Mamata Dental College and hospital, khammam, Telangana, India

**Keywords:** Polymethyl methacrylate (PMMA), denture base, flexural strength, polyethylene fibers, polypropylene fibers, reinforcement

## Abstract

Although it lacks sufficient flexural strength, polymethyl methacrylate (PMMA) finds extensive application as a foundation material for dentures. Flexural strength of heat-cured PMMA was examined in an *in vitro* experiment by adding polyethylene and polypropylene fibers. An evaluation was conducted on 120 controlled specimens by means of a three-point bending test. Results showed significant improvement with both fibers, with polypropylene (169.61 ± 16.30 MPa) providing superior reinforcement compared to polyethylene (129.29 ± 5.00 MPa) and control (99.91 ± 3.79 MPa) (p < 0.001). Based on these results, reinforcing PMMA dentures with polypropylene fibers is a good way to increase their mechanical performance and lifespan.

## Background:

Among prosthodontic materials, polymethyl methacrylate (PMMA) has been the gold standard for denture bases since its debut in 1937 [1]. The mechanical qualities of PMMA, especially its flexural strength and impact resistance, have some limits, despite its attractive attributes like biocompatibility, simplicity of production, cost-effectiveness, and great aesthetics [2]. The majority of clinical failures in maxillary full dentures are caused by these constraints, with midline fractures being the most prevalent problem [3]. Incorporating metal wires, mesh, and other kinds of fibers are among the reinforcing strategies that have been tried to enhance the mechanical qualities of PMMA denture bases [4]. Fiber reinforcing is one of these technologies that have been getting a lot of attention because of its great benefits in enhancing mechanical qualities without sacrificing aesthetics or making it difficult to fabricate [5]. The reinforcing effects of several fibers on PMMA have been studied [6]. These fibers include glass, carbon, aramid, nylon, and polyethylene. Enhancing PMMA's mechanical characteristics using polyethylene fibers has shown encouraging outcomes. These fibers are almost undetectable in denture base acrylic resins due to their low density, high modulus of elasticity, biocompatibility, and outstanding aesthetics [7]. Research has shown that PMMA's impact and flexural strengths are greatly enhanced when polyethylene fibers are included [8, 9]. The use of polypropylene fibers has also been suggested as a way to strengthen PMMA denture bases. A thermoplastic polymer, polypropylene has great qualities such as being inexpensive, chemically inert, having a low density of 0.91 g/cm^3^, and being very elastic and strong under tension [10]. Because of their smooth finish and high biocompatibility, these fibers are ideal for use in the mouth [11. Denture base resins made of PMMA may have their impact and flexural strengths increased by adding polypropylene fibers, according to previous research [12, 13]. Although both polyethylene and polypropylene fibers have shown individual promise in reinforcing PMMA, limited comparative studies have been conducted to evaluate their relative effectiveness. Most existing studies have focused on comparing these fibers with other types of reinforcements or with unmodified PMMA, but direct comparisons between polyethylene and polypropylene fibers are scarce [14, 15]. Furthermore, the optimal concentration and length of these fibers for maximum reinforcement effect remain to be established. Therefore, it is of interest to assess the flexural strength of heat cure acrylic resin with and without polyethylene and polypropylene fibers, with the hope of informing future clinical applications in prosthodontics.

## Materials and Methods:

Study Design: An *in vitro* comparative study was conducted to evaluate the flexural strength of heat cure PMMA denture base material reinforced with polyethylene and polypropylene fibers.

## Sample Size:

A total of 120 PMMA specimens were fabricated and divided into three groups (n=40 per group):

[1] Group 1 (Control): Unmodified PMMA specimens

[2] Group 2 (Test group 1): PMMA specimens reinforced with 2.5wt% polyethylene fibers

[3] Group 3 (Test group 2): PMMA specimens reinforced with 2.5wt% polypropylene fibers

## Inclusion criteria:

Only heat cure PMMA denture base material (DPI, Dental Products of India) was used in this study." Polyethylene and polypropylene fibers (Reliance India Ltd, India) of 6mm length were selected for reinforcement.

## Exclusion criteria:

Auto-polymerized, light-polymerized, or microwave-polymerized acrylic resins were excluded. Fibers of lengths other than 6mm and concentrations other than 2.5wt% were not included.

## Specimen fabrication:

Metal specimen analogues of dimensions 65mm x 10mm x 3mm (according to ISO 1567 standards) were lathe machined and used to create molds for PMMA specimens.

## For Group 1 (Control):

In brass varsity metal flasks, the metal equivalents were immersed in tooth stone. The flask halves were separated and the metal mimics were removed before the sodium alginate separating media was added. Using an acrylizer at 74°C for 2 hours and terminal boiling at 100°C for 1 hour, a mixture of 47.5g of polymer and 19ml of monomer was heated to cure PMMA, as per the manufacturer's instructions. The mixture was then packed into the mold spaces. We recovered, completed, and polished the specimens once they cooled.

## For Group 2 (Polyethylene fibers):

This group followed the same steps as Group 1, with the exception that they soaked 2.5wt% polyethylene fibers (6mm length) in monomer for 10 minutes, squeezed out the excess liquid, and then mixed the fibers with the polymer powder before adding monomer. Before being packed into the mold areas, the slurry was mixed using a vortex machine to ensure even dispersion. For Group 3 (Polypropylene fibers): The procedure was identical to Group 2, but with polypropylene fibers instead of polyethylene fibers. One week before to testing, all specimens were placed in distilled water and left at room temperature.

## Testing procedure:

The Instron AG-X, universal testing equipment, was used to measure flexural strength using a three-point bending test. A 50 mm span was used to hold each specimen, and a 2 mm/min crosshead was used to provide a vertical force at the specimen's middle until fracture occurred.

## Statistical analysis:

We used SPSS 22.0, the Statistical Package for the Social Sciences, to analyze the data. We computed group-specific descriptive statistics, including means and standard deviations. The three groups' mean flexural strength values were compared using one-way ANOVA. The Tukey's post hoc test was used to do multiple comparisons. The significance threshold was established at p < 0.05.

## Results:

We manufactured and evaluated the flexural strength of 120 PMMA specimens. Table 1 (see PDF) shows the average flexural strengths and their respective standard deviations for all groups. The results showed that all groups had statistically significant differences in mean flexural strength values (p < 0.001). Group 3 (PMMA with polypropylene fibers) exhibited the highest mean flexural strength (169.61 ± 16.30 MPa), followed by Group 2 (PMMA with polyethylene fibers) with a mean flexural strength of 129.29 ± 5.00 MPa. Group 1 (unmodified PMMA) had the lowest mean flexural strength of 99.91 ± 3.79 MPa. Multiple comparisons using Tukey's post hoc test revealed significant differences between all pairs of groups (Table 2 - see PDF). The mean difference in flexural strength between Group 1 and Group 2 was 29.38 MPa (p < 0.001), between Group 1 and Group 3 was 69.70 MPa (p < 0.001), and between Group 2 and Group 3 was 40.32 MPa (p < 0.001). Detailed flexural strength values for each specimen in all groups are presented in Table 3 (see PDF). The findings showed that the flexural strength of PMMA denture base material was greatly enhanced by both polyethylene and polypropylene fibers, however the reinforcing impact of polypropylene fibers was more noticeable than that of polyethylene fibers.

## Discussion:

This research analyzed the effects of adding polyethylene and polypropylene fibers to heat-cured acrylic resin, specifically looking at its flexural strength. While both kinds of fibers enhanced PMMA's flexural strength, the findings showed that polypropylene fibers reinforcing effect was superior to that of polyethylene fibers. The value of 99.91 ± 3.79 MPa for the mean flexural strength of unmodified PMMA (Group 1) in this research is in agreement with other findings [16, 17]. The appropriateness of the material employed in this investigation is confirmed by the fact that this value fits within the range of flexural strength criteria (≥ 65 MPa) provided by ISO 20795-1:2013 for denture base polymers [18]. A notable improvement of 29.4 percent over the control group was seen in the flexural strength (129.29 ± 5.00 MPa) after the incorporation of polyethylene fibers (Group 2). Previous research has shown that PMMA with polyethylene fiber reinforcement has improved mechanical characteristics, which is consistent with our findings [19, 20]. Due to their high modulus of elasticity, polyethylene fibers efficiently transmit stress into the PMMA matrix, therefore strengthening the material [21]. In addition, the fibers in this research were pre-treated with monomers, which probably increased the interfacial adhesion between the resin matrix and the fibers, which in turn contributed to the greater flexural strength [22]. The control group had a 69.8% increase in flexural strength (169.61 ± 16.30 MPa), whereas Group 3 showed the most notable gain, thanks to the use of polypropylene fibers. The reasons why polypropylene fibers outperform polyethylene fibers are many. Fibers made of polypropylene are stiffer and have a higher tensile strength than those made of polyethylene [23]. The fact that polypropylene melts at temperatures between 160 and 170 degrees Celsius, as opposed to polyethylene's 115 to 135 degrees Celsius, suggests that it may be more stable when heat-cured with PMMA [24, 25, 26, 27-28]. Among the two fiber types evaluated, polypropylene- reinforced PMMA appears to offer the best resistance to flexural forces, making it a promising option for clinical applications. The limits of the research must be recognized, however. To begin with, there are a lot of variables in the oral environment that could affect the performance of denture base materials, such as moisture, temperature variations, and dynamic loading conditions, which the *in vitro* examination cannot completely reproduce [29]. Second, the evaluation only included one fiber length (6 mm) and one concentration (2.5 wt%); other parameters may have produced different outcomes [30]. Thirdly, additional mechanical parameters including surface hardness, fatigue resistance, and impact strength were not evaluated in the research; it only looked at flexural strength [31]. Future research should address these limitations by conducting long-term clinical trials, evaluating different fiber concentrations and lengths, and assessing a broader range of mechanical properties. Research on how fiber orientation affects mechanical qualities and how surface treatments affect fiber-matrix adhesion would also be very beneficial [32]. Finally, heat-cured PMMA denture base material considerably increased its flexural strength when reinforced with either polyethylene or polypropylene fibers; however, the reinforcing effects of polypropylene fibers were more pronounced. These findings suggest that polypropylene fiber reinforcement could be an effective strategy to enhance the clinical performance of PMMA dentures, potentially reducing the incidence of denture fractures and improving patient satisfaction.

## Conclusion:

The flexural strength of PMMA denture base material was greatly improved by adding polyethylene and polypropylene fiber reinforcement; the impact of the former was more noticeable. The superior reinforcement provided by polypropylene fibers suggests a promising strategy to reduce denture fractures in clinical practice. Overall, polypropylene fiber-reinforced PMMA may improve the durability and longevity of prosthetic rehabilitations.

## References

[1] Vijaysinh Mori H (2023). Cureus..

[2] Kannaiyan K (2020). J Pharm Bioallied Sci..

[3] Yerliyurt K (2022). Polymers (Basel)..

[4] Ajmal BM (2023). J Pharm Bioallied Sci..

[5] Deb S (2020). J Contemp Dent Pract..

[6] Bedrossian EA (2019). J Prosthet Dent..

[7] Al-Harbi FA (2019). J Prosthodont..

[8] Mathew M (2018). J Indian Prosthodont Soc..

[9] Choksi RH, Mody PV (2016). J Indian Prosthodont Soc..

[10] Psarri C, Kourtis S (2020). J Esthet Restor Dent..

[11] Al-Dwairi ZN (2020). J Prosthodont..

[12] Kumar GV (2016). J Contemp Dent Pract..

[13] Gad MM (2016). Int J Nanomedicine..

[14] Alhotan A (2021). Materials (Basel)..

[15] Galav A (2017). Contemp Clin Dent..

[16] Hamid SK (2021). J Contemp Dent Pract..

[17] AlQahtani M (2020). Medicina (Kaunas)..

[18] Gonçalves NI (2020). J Mech Behav Biomed Mater..

[19] Gad MM (2020). Dent Mater J..

[20] Gad MM (2017). Int J Nanomedicine..

[21] Yi M (2016). Mater Sci Eng C Mater Biol Appl..

[22] Arslan M (2018). Int J Comput Dent..

[23] Arun Kumar C (2019). J Contemp Dent Pract..

[24] Zidan S (2020). Materials (Basel)..

[25] Gad MM (2019). Int J Dent..

[26] Gad MM (2018). Dent Mater J..

[27] Aldegheishem A (2021). Polymers (Basel)..

[28] Li BB (2016). Dent Mater J..

[29] Albasarah S (2021). J Adv Prosthodont..

[30] Choi JJE (2020). J Mech Behav Biomed Mater..

[31] Nakhaei M (2016). J Contemp Dent Pract..

[32] Mowade TK (2012). J Adv Prosthodont..

